# Rab GTPases in Osteoclastic Endomembrane Systems

**DOI:** 10.1155/2018/4541538

**Published:** 2018-08-15

**Authors:** Michèle Roy, Sophie Roux

**Affiliations:** Division of Rheumatology, Faculty of Medicine, University of Sherbrooke, 3001, 12th Avenue North, Sherbrooke, PQ, Canada

## Abstract

Osteoclasts (OCs) are bone-resorbing cells that maintain bone homeostasis. OC differentiation, survival, and activity are regulated by numerous small GTPases, including those of the Rab family, which are involved in plasma membrane delivery and lysosomal and autophagic degradation pathways. In resorbing OCs, polarized vesicular trafficking pathways also result in formation of the ruffled membrane, the resorbing organelle, and in transcytosis.

## 1. Introduction

Bone remodeling is required for development and growth, for mechanical adaptation, repair, and mineral homeostasis. It relies on the coupling of bone resorption followed by the formation of new bone. These interrelated processes are performed by bone-resorbing osteoclasts (OCs), which are derived from the monocyte-macrophage lineage, and bone-forming osteoblasts, which are of mesenchymal origin. Skeletal homeostasis depends on strict control over numbers of active OCs at any particular site, requiring a balance of OC proliferation and apoptosis to modulate bone turnover [1]. Bone resorption has been associated with dynamic membrane processes that are regulated by small GTPases of the Rab family and by their regulators and effectors. OCs play major roles in the coupling of bone formation and resorption by releasing growth factors from degraded matrix after transcytosis and by producing osteoblast-stimulating factors [2].

## 2. Basics of Osteoclast Biology

Osteoblasts and stromal cells support OC differentiation and activation, predominantly via macrophage colony-stimulating factor (M-CSF) and receptor activator of NF-*κ*B ligand (RANKL) pathways. These endocrine factors are produced by osteoblasts and stromal cells in the vicinity of OCs and are requirements of differentiation, survival, and bone-resorbing activities [3, 4]. Fine-tuning of bone resorption also involves osteoprotegerin, which is a secreted decoy RANKL that competes with RANK and inhibits OC differentiation and bone resorption.

### 2.1. RANKL Signaling

In precursors and mature OCs, interactions between RANKL and RANK trigger signaling cascades that activate NF-*κ*B and NFATc1. These transcription factors induce genes that have been associated with OC differentiation, survival, and activity, including those encoding integrins (*β*3-integrin), proteases (cathepsin K), and vacuolar H^+^-ATPases (V-ATPases), which are involved in bone resorption [3]. The RANKL membrane signaling complex requires recruitment of TNF receptor-associated factor 6 (TRAF6), which plays dominant roles in OC differentiation and activation and is necessary for activation of NF-*κ*B by RANKL [5]. TRAF6 also forms complexes with TGF*β*-activated kinase 1 (TAK1) and the adaptor proteins TAB1 and TAB2 [6], leading to phosphorylation of NF-*κ*B-inducing kinase with subsequent activation of the I*κ*B kinase complex and expression of NF-*κ*B. RANKL-induced TRAF6 recruitment also activates the PI3K/Akt pathway and MAP kinases, including p38, ERK, and JNK, and is terminated by ubiquitination of TRAF6 and autophagic and proteasomal degradation of the signaling complex [7]. The p62 scaffolding protein (sequestosome1) provides a functional link between RANKL and TRAF6-mediated NF-*κ*B activation and has been shown to propagate RANKL-activating signals [8, 9]. Among binding domains of p62, the ubiquitin-associated (UBA) domain interacts with polyubiquitinated proteins and mediates their degradation via autophagic or proteasomal pathways [10], which are reportedly central to OC activities [11, 12].

### 2.2. M-CSF Signaling

Interactions between M-CSF and its receptor c-fms induce receptor dimerization and activate its intrinsic tyrosine kinase activity. Subsequent autophosphorylation of intracellular c-fms domains leads to the recruitment and docking of PTB- (phosphotyrosine-binding) and SH2- (Src homology region 2) domain-containing effector proteins and activation of the signaling pathways that promote OC proliferation, survival, and motility. Specifically, activation of PI3K/Akt and ERK1/2 pathways regulates proliferation of OC precursors and OC survival. In addition, small GTPases have been associated with c-Src signaling, which regulates F-actin ring formation and podosome dynamics. M-CSF signaling ends by receptor internalization and subsequent degradation by proteases and lysosomes [4, 13].

### 2.3. Osteoclastic Bone Resorption

**Figure 1:**
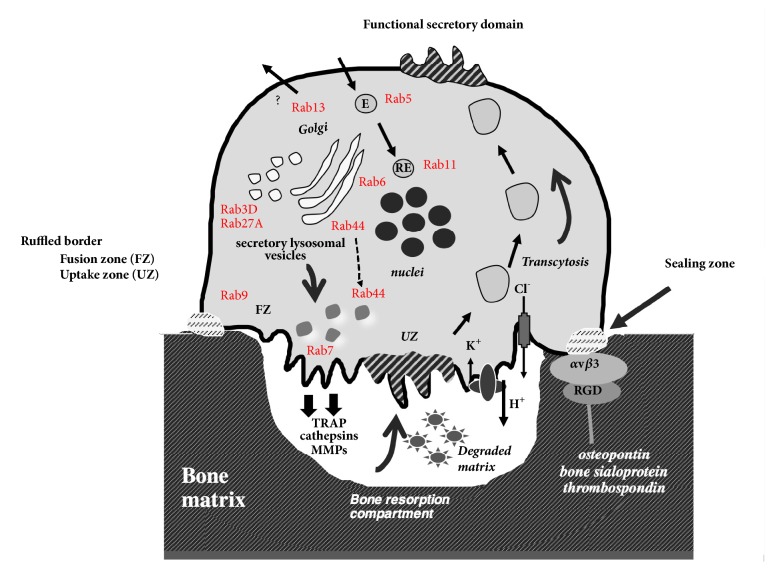
**Bone resorption and transcytosis. **On adhesion to the bone, OCs become polarized and reorganize their cytoskeleton. The sealing zone (or organelle-free clear zone) is formed by a peripheral belt of adhesive structures that delimit the ruffled border, which is a highly specialized area consisting of membrane expansions directed toward the target bone surface. The ruffled border is formed by polarized vesicular trafficking and plays a critical role in the degradation of bone matrix through acidification by V-ATPases and proteases released by the fusion of secretory lysosomal vesicles. The central ruffled membrane represents the uptake zone where degraded bone matrix products are internalized and transported by transcytosis toward the functional secretory domain. Several Rab proteins identified in OCs have been added (in red). Rab7 plays a direct role in the ruffled border formation. Rab3D and Rab27A have been involved in the trafficking of secretory vesicles essential for bone resorption.* E: endosome *and* RE: recycling endosomes.*

OCs are large multinucleated cells formed by fusion of mononuclear precursors. These cells are highly motile and alternate between migratory and bone-resorbing stages, with dramatic phenotype changes. Following adhesion to bone, the polarized OC reorganizes its cytoskeleton to form specialized zones: sealing zone, ruffled border, functional apical secretory domain, and basolateral domains [14]. The sealing zone is formed by densely packed actin-rich podosomes that delimit the ruffled border, which is a highly specialized area consisting of membrane expansions that are directed toward the target bone surface. The fusion zone of the ruffled membrane mediates polarized vesicular trafficking and plays critical roles in the degradation of bone matrix through acidification by V-ATPases and proteases that are released upon fusion of secretory lysosomal vesicles. In contrast, the central uptake zone mediates internalization of degraded bone matrix products, followed by transcytosis toward the functional secretory domain that releases degradation products into extracellular environment [15–17]. In nonresorptive or migrating OCs, sealing zones reform into podosome belts and relaxed OCs are depolarized [18]. During bone remodeling, bone resorption ceases with apoptosis of OCs prior to bone formation [8, 19, 20] (Figure 1).

### 2.4. Endomembrane Systems in Osteoclasts

As in other eukaryotic cells, OCs present in their cytoplasm membrane-bounded organelles and vesicles, such as endoplasmic reticulum (ER), Golgi apparatus, lysosomes, and endosomes, which are major conserved pathways of the endomembrane system. These structures are mainly involved in the biosynthesis and turnover of surface molecules, as well as the uptake and degradation of components of the cell environment, grouping the endocytic and exocytic trafficking pathways [21]. Other lysosomal-bound organelles are present in resorbing OCs and include secretory lysosomal vesicles that contribute to the formation of the ruffled membrane, forming the resorptive organelle. Vesicular transport is also critical for autophagy and related mechanisms of lysosome-mediated protein degradation. The conserved endomembrane system and lysosome-related organelles found in active OCs are both regulated by Rab GTPases.

## 3. Rab GTPases and Their Regulators

### 3.1. Small GTPases

Small GTPases of the Ras superfamily include Ras, Rho, Ran, Arf, and Rab subfamilies [24, 25], which regulate essential intracellular pathways for cell morphology and signaling. Members of the Ras family are known to regulate cell proliferation, differentiation, and apoptosis [25]. In OCs, Ras GTPases have been associated with M-CSF signaling and promote OC proliferation and survival via the ERK pathway [13]. In contrast, members of the Rho family regulate actin cytoskeletal remodeling during OC polarization. Among these, RhoA, Rac1, Cdc42, and RhoU are essential for assembly and disassembly of podosomes and for the formation of F-actin rings [26]. Ran proteins are the most abundant among Ras superfamily GTPases, and although their roles in OCs remain uncharacterized, they have been associated with nuclear transport in other cell types [25]. Members of the Arf subfamily of GTPases mediate vesicle transport through interactions with coat complexes that cover vesicles and by recruiting lipid-modifying enzymes to membranes. Among these, Arf6 is the only protein with known functions in OCs and has been shown to interact with Rho proteins to regulate the formation of sealing zones. Arf6 may also participate in lysosomal trafficking through interactions with Rac1 and Rab7 [26].

Rab family are essential components of cell and organelle membranes and regulate vesicular trafficking during endocytosis, exocytosis, and other vesicular changes [27]. Because vesicular transport is critical in OCs not only for autophagy but also for the essential bone resorption functions, we reviewed mammalian Rab GTPases with known roles in the regulation of vesicle trafficking during autophagy and summarized associations of Rab proteins with bone resorbing functions of OCs.

### 3.2. Rab GTPases

Rab proteins form the largest subfamily of small GTPases, and about 70 Rabs have been identified in the human genome [22]. However, although Rab proteins play a major role in cell homeostasis and numerous Rabs have been now identified, functions of most of them remain unclear. Rabs regulate various stages of vesicular transport, including membrane budding, formation of transport vesicles, movement of vesicles along cytoskeleton, and membrane or vesicle fusion, and interact with numerous sorting adaptors, tethering factors, kinases, and phosphatases [28]. Active Rabs predominantly integrate and mediate intracellular trafficking signals by recruiting various effectors to define distinct microdomains on membrane surface for regulatory molecules and downstream effectors [29]. Although most Rab GTPases are ubiquitous, they have been grouped into subfamilies according to localization in cellular compartments, where they function with organelle specificity [30]. However, differing functions of some Rab proteins have been shown in differing cell types. In particular, Rab13 is involved in glucose transporter traffic in muscle cells but not in OCs [31], and Rab27A is specifically expressed in secretory cells [32].

### 3.3. Guanine–Nucleotide Exchange Factor (GEF)/GTPase-Activating Protein (GAP): Critical Regulators of Rab GTPase Cycles

**Figure 2:**
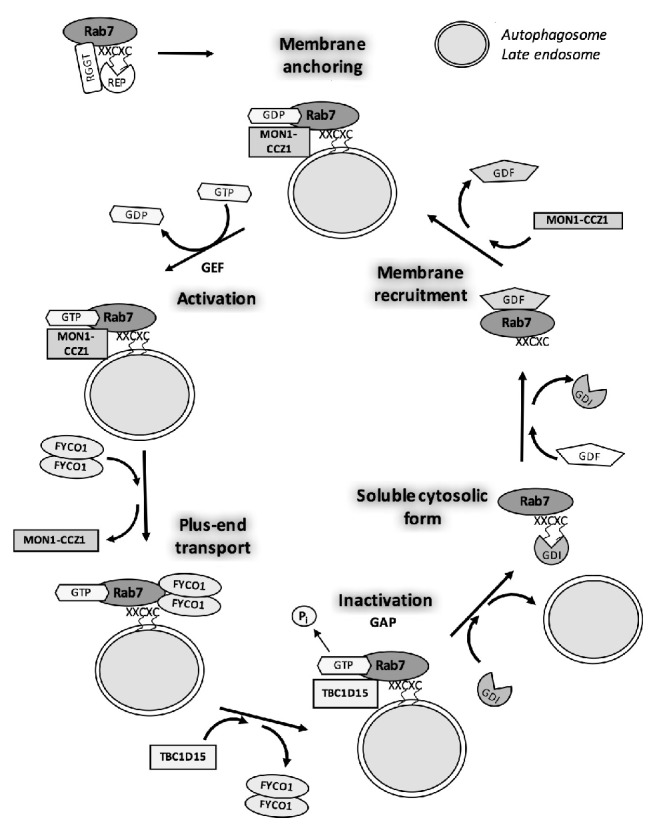
**Rab GTPase regulating cycle: example of Rab7. **Rab7 is required for the transfer of the cargo from late endosomes and autophagosomes to lysosomes. Like other Rabs, Rab7 carboxy-terminus contains motif such as XXCXC, in which the two cysteines are substrate for prenylation, a lipid transfer crucial for membrane anchoring. During the maturation of endosomes, Rab5 is exchanged with Rab7, a process facilitated by the protein complex Mon1-Ccz1, a Rab7 GEF. Membrane-anchored GDP-Rab7 is then activated to GTP-Rab7 by this GEF. The activation allows the release of Mon1-Ccz1 and the recruitment FYCO1, a Rab7 effector protein, which mediates Rab7 actions, such as autophagosome transport trough microtubule at the plus-end. TBC1D15 RabGAP facilitates the inactivation of Rab7. GDP-Rab7 is recognized by a GDI and released into the cytosol to its soluble form. The soluble GDP-Rab7 will eventually be recognized by GDF, driving the GTPase to a novel vesicle to start a new cycle (adapted from [22, 23]).* Abbreviations: GAP: GTPase-activating protein; GDI: GDP dissociation inhibitor; GEF: guanine–nucleotide exchange factor; REP: Rab escort protein; RGGT: Rab Geranyl-Geranyl transferase; FYCO1: FYVE-coiled coil containing protein.*

Similar to all members of the Ras superfamily, Rab proteins receive C-terminal posttranslational modifications from the mevalonate pathway, such as prenylation with the lipids farnesyl pyrophosphate (FPP) and geranylgeranyl pyrophosphate (GGPP) [33]. These posttranslational modifications add one or, for most Rab GTPases, two GGPP groups and are required for appropriate localization to cytoplasmic surfaces of membranes and vesicles [22]. The Rab escort protein (REP) facilitates the prenylation by forming a stable complex with the Rab geranyl-geranyltransferase (RGGT) and chaperones newly geranylgeranylated Rab to the target membrane. A second conserved region among Ras proteins enables transitions between the active GTP-bound state that recruits effectors and the inactive GDP-bound state. In addition, a GDP dissociation inhibitor (GDI) has been shown to recognize inactive forms of Rab GTPases at membranes and binds lipophilic groups to solubilize Rab proteins for release into the cytosol under basal conditions. Subsequently, a GDI displacement factor (GDF) mediates delivery of Rab GTPases to specific target membrane structures, and the release of GDI and positioning of GTP are catalyzed by guanine–nucleotide exchange factors (GEFs), which activate Rab proteins. Active Rab GTPases can then interact with effectors to fulfill their functions. Subsequently, Rab GTPases hydrolyze GTP to GDP after binding to a GAP, which facilitates the reaction and, in the presence of GDI, inactive Rabs are finally released into the cytosol [24, 34] (Figure 2).

At least six types of Rab-activating GEFs have been characterized including DENN-, Vps9-, and Sec2-domain containing proteins and multisubunit TRAPP complexes, which are not structurally related [24]. At least 40 putative Rab GEFs are expressed in humans, and some of these have specific Rab protein partners, whereas others activate several Rabs. Moreover, Rab proteins are activated by multiple Rab GEFs of diverse types [35]. Almost all Rab GAPs contain a highly conserved TBC domain (TBC/RabGAPs) that accelerates hydrolysis of GTP to inactivate Rab proteins. In addition to the 44 mammalian proteins of the TBC/RabGAP family, the Rab3GAP complex, which targets Rab3, is the only RabGAP without a TBC domain [36]. Moreover, TBC/RabGAPs are less diverse than their Rab targets, and each may inactivate multiple Rabs [37]. Similarly, multiple TBC/RabGAPs regulate single cellular events by inactivating various Rab proteins at distinct steps of the process. In contrast, single TBC/RabGAPs can modulate numerous cellular events by inactivating single Rab proteins that participate in multiple processes. Interactions with TBC/RabGAP or RabGEF may not regulate the GDP/GTP state of the binding Rab protein, and some GAPs and GEFs can be recruited by Rabs to regulate the GDP/GTP state of neighboring Rab GTPases [35, 38]. Currently, only one RabGEF, RIN3 [39] and one TBC/RabGAP, TBC1D25, have been reported to be expressed in human OCs [40], although the function of both regulators remains unknown in these cells.

### 3.4. Rab GTPase Effectors

Activation of Rab GTPases by GEFs induces conformational changes allowing the recruitment of specific soluble effector proteins, to coordinate vesicular trafficking. As for the other GTPases, the Rab protein structure contains two regions close to the guanine nucleotide, switch I and switch II, which undergo conformational changes upon nucleotide exchange (GDP/GTP) allowing the recruitment of effector proteins that interact with active GTP-Rabs selectively at these regions [30, 34]. Effectors rarely bind to inactive Rabs, such as protrudin that interacts with GDP-Rab11 [41]. They might also interact independently of the GTP/GDP status, such as LC3 interacting with Rab12 in a domain probably distinct from switch I and switch II [42]. Rab GTPases with their multiple effectors can create various microdomains on the membrane surface to coordinate vesicle trafficking. A single Rab can interact with different effectors at different sites to participate in several steps of vesicular traffic. A single effector protein can also interact with two Rab GTPases to bring membranes and effectors closer together [29].

The effectors of Rab GTPases are involved in the movements of organelles and vesicles and contribute to the specificity of target membranes. They include sorting adaptors, tethering factors, motor proteins, but also kinases and phosphatases [28, 29, 43]. The Rab GTPases therefore appear to play multiple roles. For example, among the known Rab5 effectors are Cczl which is a Rab7 GEF; Vps34 which is a kinase involved in autophagy; Kif13A which is a motor protein involved in plus-end microtubule transport [43].

## 4. Roles of Rab GTPases and Their Regulators in Autophagy

Numerous Rab proteins and regulators have been associated with autophagy, although these data indicate ever-increasing complexity of Rab protein interactions in autophagic pathways.

### 4.1. Autophagy Basics

**Figure 3:**
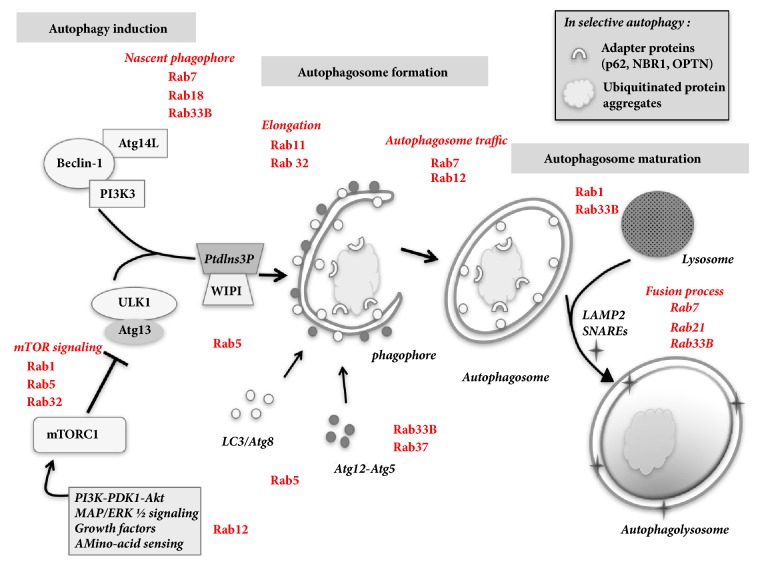
**Autophagy basics. **Autophagy proceeds by a series of ordered events controlled by a group of autophagy-related (Atg) proteins coordinating 3 major steps: (1) the initial induction and nucleation of autophagic vesicles require the activity of specific complexes, including the PI3K complex, composed of type-III PI3K and Beclin1, and the ULK1 complex. Other upstream signaling pathways regulate ULK1 complex activity, including PI3K/Akt and ERK, which work through mTORC1, a potent inhibitor of autophagy which is sensitive to rapamycin. (2) Vesicle expansion and completion of the autophagosome: this step requires the products of 2 ubiquitin-like conjugation systems that produce Atg5-Atg12 and Atg8/LC3-PE. (3) Finally, the mature autophagosome fuses with a lysosome through a step that involves proteins such as Rab7 and SNAREs. Rab GTPases that have been involved in the different steps of autophagy are indicated (in red).* Abbreviations: Beclin1: BCL-2 interacting myosin/moesin-like coiled-coil protein 1; LC3: light chain 3 [Atg8 (yeast) is called LC3 in mammals]; mTORC1: mammalian target of rapamycin complex 1; PtdIns3P: phosphatidylinositol 3-phosphate; SNAREs: N-ethylmaleimide-sensitive factor attachment protein receptors; ULK1: UNC51-like kinase 1; WIPI: WD repeat domain phosphoinositide-interacting protein; p62: sequestosome 1; NBR1: neighbor of BRCA1 gene 1; OPTN: optineurin; LAMP2: lysosome-associated membrane protein 2.*

Macroautophagy is a trafficking pathway that delivers cargo in double-membraned autophagosomes to the lysosomes for degradation and recycling. Autophagy takes place in all cells, where it maintains cell homeostasis or, in response to stress, starvation or hypoxemia, eliminates damaged components, and provides cells with energy and nutrient resources. Autophagy-related proteins coordinate the three main steps of autophagy as follows:** (1)** the initial induction of a membrane core requires the activity of 2 complexes: the PI3K complex composed of type-III PI3K plus the Beclin 1 protein, and the ULK1 complex, which congregate at phagophore assembly sites to initiate autophagy. These pathways are interconnected via PtdIns(3)P complexes, which signal via PtdIns(3)P-binding effectors that are specific to autophagy.** (2)** Vesicle expansion then leads to the formation of mature autophagosomes, at first requiring recruitment of conjugated Atg5-12/16L complexes to phagophores during elongation. Cytoplasmic LC3-I is then conjugated to phosphatidylethanolamine (PE) and generates LC3-II, which is present on interior and exterior surfaces of forming vesicles.** (3)** Finally, mature autophagosomes fuse with late endosomes or lysosomes to produce autophagolysosomes that degrade their contents [44, 45]. Autophagosomes may also fuse with plasma membranes to release their contents into extracellular spaces [46] (Figure 3).

Autophagy is regulated by growth factors, amino acids, glucose, and energy status, and these upstream signals are integrated through the mammalian target of rapamycin complex 1 (mTORC1), which is a potent multiprotein inhibitor of autophagy that comprises the mTOR catalytic subunit and the regulatory associated protein of mTOR (raptor). Inhibition of mTORC1 following starvation or exposure to rapamycin leads to activation of ULK1 and induction of autophagy [47]. Moreover, after interacting with raptor, scaffold p62 provides an alternative docking site from which mTORC1 can be regulated [48]. During selective autophagy, ubiquitinated proteins are recognized by the UBA domain of adaptor proteins such as p62, NBR1 (neighbor of BRCA1 gene 1), and optineurin, and these are referred to as sequestosome-1-like receptors. These adaptors target ubiquitinated proteins to nascent LC3-presenting autophagosomes via their LC3-interacting regions [49].

The early stages of autophagosome formation (phagophore), elongation through vesicular fusion, and maturation (autophagolysosome) are dependent on the supply of membranes with appropriate properties, suggesting autophagic roles of vesicle trafficking proteins, such as Rabs and their regulators [36].

### 4.2. Osteoclast Autophagy

Currently, few studies report roles and regulatory parameters of autophagy in OCs. However, in murine and human OC-like cells, autophagy has been shown to be involved in oxidative stress- and hypoxia-induced differentiation [12, 50]. Moreover, inhibition of autophagy by mTORC1 promoted OC survival and RANKL-induced formation and activation of OCs [51, 52]. Dysfunctional autophagy has also been identified in the OC phenotype of Paget's bone disease, in which defects in autophagy induction and clearance of autophagosomes have been observed [53], and activated autophagy in OCs was associated with rheumatoid arthritis [54].

### 4.3. Rab Small GTPases That Are Associated with Autophagy

During autophagy, Rab GTPases regulate vesicle tethering, transport, and fusion, and several Rabs (Rab1, Rab5, and Rab32) have been associated with autophagosome maturation, phagophore expansion (Rab11), and fusion with lysosomes (Rab7), and some regulate both autophagosome initiation and maturation (Rab33B). Moreover, the Rab proteins Rab8B, Rab9A, Rab23 [46, 55], Rab24 [56], Rab30 [57], and Rab35 [58] have been implicated in xenophagy (antimicrobial defense) and mitophagy (mitochondrial autophagy), which are beyond the scope of the present review.

Following identification of Rab GTPases that play roles in autophagy, their regulators RabGAPs and GEFs and various effector proteins were discovered. Among RabGAPs, 12 TBC/RabGAPs and a non-TBC Rab3GAP complex have been studied in the context of autophagy [36]. Moreover, some TBC/RabGAPs are considered autophagic because they interact with Atg8/LC3 proteins, although their precise functions have not been established. In contrast, some TBC/RabGAPs may inhibit autophagy by inhibiting LC3-II expression, as shown in an overexpression screening study [59]. In particular, TBC1D5 has no known Rab target in autophagy but interacts with LC3 and facilitates trafficking of Atg9 and active ULK1 to autophagic structures [59, 60]. Multiple GEFs and Rab effector proteins have also been involved in the autophagic process, and target Rabs have been identified for TBC/RabGAPs, GEFs, and various effector proteins, as detailed below.

Autophagic functions have been described for many Rab GTPases, and these are predominantly related to initial induction of the membrane core. In particular, Rab5 has been implicated in the regulation of mTORC1 signaling, conjugation of Atg5 and Atg12, and in the production of PtdIns(3)P at the phagophore through recruitment of type-III PI3K [55]. Rab1 and Rab32 have also been associated with mTORC1 signaling and in membrane trafficking from the ER, allowing phagophore expansion [55]. Although the involvement of Rab32 in autophagy is evident only from its localization with LC3 on vesicles, the mechanisms and related effectors remain unknown. Conversely, the function of Rab1 is better understood, and recruitment of the Rab1 GEF TRAPPIII to autophagosomes by Atg17 has been shown to activate Rab1. GTP-Rab1 then recruits p115 and induces vesicle budding from the ER [46]. TBC1D20 was also identified as a GAP for Rab1 and is reportedly required for autophagosome maturation [61].

Rab11 participates in membrane trafficking from the ER and has been implicated in phagophore maturation [55]. In HEK293 cells, TBC1D14 was shown to act as a Rab11 effector, but no GAP activity was observed. TBC1D14 forms a complex with ULK1 and was colocalized with Rab11, and these interactions were required for the transport of recycling endosomes to sites of phagophore formation, and for starvation-induced autophagy [62]. Furthermore, TBC1D14 has been shown to interact with Rab1 GEF TRAPPIII, facilitating the switch from Rab11^+^ to Rab1^+^ vesicles and rapid recycling of Atg9 for autophagosome formation [63].

Rab12 reportedly played an indirect role in the induction of autophagy and a direct role in autophagosome trafficking. Initially, Rab12 was shown to regulate the initiation of autophagy by affecting upstream signals for mTORC1 activation independently of Akt. Rab12 activities were also correlated with intracellular concentrations of amino acids, reflecting degradation of the amino-acid transporter PAT4 [64]. During starvation-induced autophagy, ULK1/2 is activated by the suppression of mTORC1 activity and mediates the phosphorylation of DENND3, which is a Rab12 GEF. Activated GTP-Rab12 is then localized to recycling endosomes and autophagosomes, where it interacts with LC3. However, the GTP/GDP state of Rab12 does not affect LC3/Rab12 interactions, indicating that LC3 is not a Rab12 effector, although after binding LC3, DENND3-induced Rab12 activation was shown to facilitate autophagosome trafficking [42].

More recently, Rab18 was characterized as a positive regulator of early autophagy stages in human fibroblasts, and changes in its expression levels were correlated with changes in autophagic flux. Moreover, this effect was dependent on the heterodimer Rab3GAP1/2, which was previously identified as a RabGEF for Rab18 [65]. Rab37 has also been implicated in phagophore formation upon induction of autophagy. Specifically, in the GTP-bound state, Rab37 reportedly interacted with Atg5 and recruited Atg5-12/16L complexes to promote elongation of isolation membranes and LC3-II expression [66, 67].

In another study, Rab33B interacted with the conjugated complex Atg5-12/16L and promoted LC3-PE conjugation after binding Atg16L specifically, thereby modulating autophagosome maturation [68]. The RabGAP TBC1D25 binds various Rabs and, among these, Rab33B is a recently discovered binding partner of LC3 that contributes to late stage autophagosome formation, which involves fusion between autophagosomes and lysosomes. In mouse embryonic fibroblasts, TBC1D25 has been shown to directly interact with LC3 on autophagosome membranes and inactivates Rab33B through its GAP activity, resulting in delayed autophagosome maturation [69].

Relatively little is known of the regulatory mechanisms of late-stage autophagy, although Rab21 was shown to contribute to the formation of autophagolysosomes. Upon starvation-induced autophagy, Rab21 was activated by the GEF MTMR13 (Myotubularin-related protein 13), which carries a DENN domain. In addition, activation of Rab21 promoted interactions with its effector VAMP8 (Vesicle Associated Membrane Protein 8), which is an R-soluble N-ethylmaleimide-sensitive factor attachment receptor (R-SNARE) that mediates membrane fusion [70]. Rab7 is predominantly involved in autophagosome fusion with endosomes and lysosomes but also contributes to other steps of autophagy. Because Rab7 plays major roles in membrane trafficking in OCs, its role will be discussed in detail below.

## 5. Rab GTPases in Osteoclasts

Bone-resorbing OCs contain two well-characterized intracellular vesicular trafficking pathways. The first involves directional transport of secretory vesicles toward plasma membranes facing the bone and their fusion to form the ruffled border. This membrane specialization is essential for bone resorption and is similar to that of lysosomal membranes, warranting wide consideration as a lysosomal-related organelle [71]. The second trafficking pathway involves transcytosis of degraded matrix products from the resorption lacuna to the apical functional secretory [14, 15]. Among numerous small GTPases, at least 26 are transcribed in human OCs [31]. Transcription of the Rab GTPases Rab1B, Rab4B, Rab5C, Rab9, Rab11B, Rab27B, and Rab35 was also demonstrated in rodent OCs but these Rabs were either not tested or not detected (Rab9, Rab27B) in human OCs [32, 72]** (Suppl Table** (available here)**)**.

In human OCs, Rab13 gene expression was shown to be highly upregulated during human OC differentiation, and although the roles of Rab13 in OCs are unclear, it does not seem involved in bone resorption, transcytosis, endocytosis, and glucose transport. Moreover, downregulation of Rab13 does not affect OC differentiation and, in mature OCs, Rab13 was localized to small vesicular structures between* trans*-Golgi networks and basolateral membranes, suggesting associations with secretory functions [31].

In rodent OCs, the Rab GTPases Rab2B, Rab3A, Rab3B/C, Rab3D, Rab5, Rab6, Rab7, Rab9, Rab10, Rab11, Rab14, Rab18, and Rab27A were detected in protein studies [32, 72–75], although their specific functions in OCs remain mostly undefined.

The expression of Rab3 isoforms with known roles in exocytosis was previously investigated in murine OC precursors, which expressed Rab3A and Rab3B/C [73]. In further studies, Rab3D knockout mice had high bone mass and impaired osteoclastic bone resorption. Moreover, OCs from Rab3D deficient mice displayed normal F-actin rings and podosome formation, but abnormal ruffled borders. Rab3D was the major OC-expressed Rab3 isoform and was associated with a nonlysosomal post-Golgi trafficking step that is required for OC bone resorption [74]. As in other cell types, subcellular localization and colocalization of Rab5C with early endosome antigen1 (EEA1) suggest that Rab5C is associated with early endosomes. Rab11B is one of the most abundant Rabs in rodent OCs and was localized with perinuclear recycling compartments. However, Rab5C and Rab11B were not localized to ruffled borders in resorbing OCs but contributed to upstream stages of resorption-related vesicular transport [72, 76]. Rab6 was highly expressed and localized in the Golgi compartment in OCs in a previous study, but no specific function was established. In addition, Rab9 partially colocalized with Rab7 in intracellular vesicles but, in ruffled borders, had complementary distributions to those of Rab7 [72]. Although Rab9 was involved in trafficking of late endosomes to the* trans*-Golgi in other cell types, its roles in OCs remain undetermined [22].

Rab27A mRNA expression increased during OC differentiation in mice, whereas decreased expression of the Rab27B isoform was observed. Rab27A is an important Rab GTPase in secretory cells, such as endocrine and exocrine cells and leukocytes, and OCs from* ashen* mice lacking Rab27A had defects in actin ring formation, irregular distributions of lysosome-associated membrane protein (LAMP2) and cathepsin K, and impaired bone resorption. These data suggest that Rab27A is involved in the transport of secretory lysosomes in resorbing OCs [32]. New data also suggest that GDP-Rab27A interacts with *α*3 subunit of the lysosomal proton pump, V- ATPase. The small GTPase could be recruited to lysosomes by *α*3 and then activated to regulate secretory lysosomes biodistribution [76]. While Rab38 mRNA expression was highly increased by RANKL in an NFATc1-dependent manner in murine OCs, it was not a significant regulator of OC formation or function [77]. The novel atypical Rab protein Rab44 was detected in mice OCs at mRNA and protein levels. This Rab was localized in Golgi complexes and lysosomes and was shown to modulate pH by mediating Ca^2+^ influx. Rab44 also inhibited OC differentiation by modulating intracellular Ca^2+^ levels and activating NFATc1 [78].

Autophagy is another vesicular trafficking process by which OCs degrade and recycle misfolded proteins and damaged organelles [79]. In addition to roles in the formation of double-membrane autophagosomes, autophagy-related proteins, such as LC3B, Atg5, and Rab7, are involved in the formation of ruffled borders and sealing zones and in the fusion of secretory lysosomes and subsequent bone resorption [80, 81]. Details of Rab7 functions in OCs are discussed below.

Transcytosis is a vesicular trafficking pathway that has been observed in resorbing OCs and involves endocytosis of degraded products at uptake zones of ruffled borders and vesicle transport to the functional secretory domain for exocytosis [15–17]. The role of Rab proteins in transcytosis has been characterized in polarized cells; in particular Rab17 has been involved in vesicular trafficking from basolateral to apical membranes in epithelial cells [26], although no Rab proteins have yet been associated with transcytosis in OCs. Moreover, while Rab11 and Rab5 were shown to be involved in early and recycling endocytosis [22], neither Rab11 nor the Rab5 effector EEA1 is localized to uptake zones of ruffled borders [15], suggesting that these Rabs are not implicated in OC transcytosis. Because OCs have unique vesicular trafficking pathways and unique functions of Rab proteins, such as those of Rab13 and Rab27A [31, 32], functions of Rab proteins in OC transcytosis cannot be assumed on the basis of their functions in other cell types. Hence, further studies of these trafficking pathways are required in resorbing OCs.

## 6. Rab7 Is Crucial for OC Activity and Autophagy

Rab7 is among the best characterized Rab proteins involved in vesicular trafficking, with evidence that it contributes to endocytic pathways that facilitate endosome maturation and that it regulates the transport of the early endosomes to late endosomes [55] and positions lysosomes by regulating cytoskeletal transport [23]. Rab7 is also known to be involved in numerous lysosome processes, including the biogenesis of phagosomes, autophagosomes, and lysosomal-related organelles. In addition, TBC/RabGAPs and GEFs have been found to regulate Rab7, whereas little is known about REP, GDI or GDF involved in the regulation of this Rab protein [23]. Most Rab7 regulators and effector proteins are related to autophagic process and vesicular trafficking during the formation of ruffled borders for bone resorption** (**Figure 2).

Taken together, previous studies indicate that Rab7 is involved in multiple steps of the autophagy process and plays direct and indirect roles in the regulation of mTORC1-mediated autophagy, in the biogenesis of nascent autophagosomes through effector protein PLEKHF1 (Phafin1), in autophagosome transport along microtubules via the effector proteins FYCO1 (FYVE-coiled coil containing 1), RILP (Rab7-interacting lysosomal protein), and ORP1L (Oxysterol-binding protein-related protein 1L), and in the fusion of autophagosomes with late endosomes or lysosomes via the HOPS (homotypic fusion and protein sorting) complex and PLEKHM1 (pleckstrin homology domain containing, family M member 1) [55, 60]. In addition, lysosomal positioning was reported to regulate mTORC1, as indicated by colocalization of mTORC1 with lysosomes at the cell periphery [82] and with lysosomal Rab7 [83] upon activation. Rab7 has also been implicated in the early stages of autophagy through its effector protein PLEKHF1 (Phafin1), which led to accumulation of autophagosome-like structures when overexpressed. Previous studies also show that Rab7 is required during the early stages of antimicrobial autophagy against* Streptococcus pyrogenes*, although the ensuing molecular mechanisms remain unknown [55]. The transport in autophagy is mediated by the activation of Rab7 and the recruitment of its effectors. Mon1-Ccz1, a Rab7 GEF, facilitates the exchange of Rab5 to Rab7 on late endosomes, and activated GTP-Rab7 may then recruit effector proteins such as FYCO1 and RILP/ORP1L. These effectors also affect Rab7 through interactions with microtubules during positioning of late endosomes and lysosomes. FYCO1 reportedly interacts with kinesin during microtubule plus-end transport of autophagosomes [46]. Conversely, both RILP and ORP1L were shown to interact with dynein and dynactin to regulate microtubule minus-end transport [23].

Finally, Rab7 was shown to be required for fusion between autophagosomes and lysosomes. Specifically, the Rab7 effector protein PLEKHM1, which is an adaptor protein that interacts with LC3 at the autophagosome, and with the HOPS complexes that include the subunit Vsp39, which is a Rab7 GEF and promotes autophagosomes–lysosome fusion [60]. Although PLEKHM1 may be an essential regulator of autophagy in HeLa cells, it may not be implicated in autophagy in OCs. Accordingly, autophagic flux was not changed in OCs from* Plekhm1−*/*− *mice, and PLEKHM1 exerted no effects on autophagy in A549 lung cells [84], suggesting that its roles in autophagy are cell specific.

Autophagic flux is also regulated by the RabGAP TBC1D2, which reportedly inhibited Rab7 and interacted with LC3 in human keratinocytes. Specifically, Rab7 was inactivated on lysosomes after LRRK1 (Leucine Rich Repeat Kinase 1) was recruited to vesicles to activate TBC1D2 [46, 85]. TBC1D2 overexpression also led to the accumulation of enlarged autophagosomes, and its depletion delayed autophagy flux [86], further indicating its regulatory roles. TBC1D15 was also identified as a Rab7 GAP during autophagy and was associated with the Atg8 protein network, but its precise role remains unknown [38, 87].

As stated above, Rab7 was strongly localized at ruffled borders in resorbing OCs from rodents and had perinuclear distributions typical of late endosomes in inactive OCs [88]. In further studies, downregulation of Rab7 in OCs impaired the formation of F-actin rings, inhibited polarization, and reduced resorption, indicating roles in vesicular trafficking during bone resorption [89].

Little is known about GAPs and GEFs regulators coordinating Rab7 activities in OCs. It has recently been published that the nonactivated form GDP-Rab7 interacts with the *α*3 subunit of the V-ATPase proton pump in OCs. This subunit is also important for localization of GTP-Rab7, and in OCs deficient in *α*3 subunit, the active form GTP-Rab7 has been shown to be diffusely expressed in the cytoplasm, whereas it is normally localized to secretory lysosomes. Taken together these results suggest that GDP-Rab7 is recruited by subunit *α*3 to secretory lysosomes and then activated by a still unknown factor, allowing GTP-Rab7 to bind effectors involved in vesicle trafficking along microtubules [76].

PLEKHM1 colocalizes with Rab7 in late endosomes/lysosomes and could be a Rab7 effector protein for OC vesicle trafficking [84, 90]. Moreover,* Plekhm1*−/− OCs had compromised resorption activity because of the absence of ruffled borders. Four PLEKHM1-interacting partners were identified in this model, and the strongest interaction was found with DEF8 (differentially expressed in FDCP 8 homolog), for which binding was enhanced by the presence of Rab7. FAM98A (family with sequence similarity 98 member A) and NDEL1 (NudE neurodevelopment protein 1 like 1) have been associated with microtubules, and their interactions with PLEKHM1 may regulate lysosome positioning. These adaptor proteins have also been shown to interact with TAK1, which is a downstream molecule of the RANKL/RANK signaling pathway, and RAP1B (RAS-Related Protein), which is involved in integrin signaling [55]. TRAFD1 (TRAF-type zinc finger domain containing 1) was also shown to interact with PLEKHM1 depending on the presence of GTP-Rab7. Downregulation of TRAFD1 also impaired trafficking and lysosomal secretion depending on the presence of PLEKHM1, leading to decreased OC bone resorption [91]. Hence, Rab7 and its effectors may mediate OC biology by recruiting proteins from various trafficking and signaling pathways of OCs.

Finally, the Rab7 effector protein Rac1 was localized at ruffled borders and was shown to interact specifically with Rab7 at sealing zones in resorbing OCs. Because only actin filaments are present at sealing zones, Rac1 may link lysosomes transported via microtubules to actin filaments, allowing transport to the plasma membranes facing the bone and formation of ruffled borders [92]. Moreover, Rac1 was shown to interact with Rab7 GAP TBC1D2 (ARMUS), indicating that it may also regulate Rab7 [86]. This study also showed that Rab7 does not interact with its effector protein RILP in resorbing OCs. Hence, this interaction may be specific to the autophagy process in this cell type. Collectively, published studies provide strong evidence that Rab7 is a crucial Rab protein for OC activity, although the ensuing mechanisms remain unclear.

## 7. Endomembrane Systems and Rab GTPases in Bone Diseases

### 7.1. Osteopetrosis

Osteopetrosis is an inherited heterogeneous bone disease that is characterized by the inability to resorb bone and consequent high bone mass and generalized osteosclerosis [93]. Multiple models of osteopetrosis have been generated in mice, and these indicate that failure of bone resorption may follow the absence of OCs, as in M-CSF-deficient op/op mice [94], c-fos-deficient mice [95], and RANK- or RANKL-deficient mice [96, 97], which all have deficiencies of OC differentiation. Because NF-*κ*B plays a key role in OC formation, p50 and p52 NF-*κ*B subunits were ablated in double knockout mice, and these animals developed a form of osteopetrosis that was caused by defects in OC differentiation [98, 99]. A defect in one of the multiple steps of bone resorption process (ruffled membrane formation, proton transport, and proteolytic enzyme secretion) may also lead to osteopetrosis, as indicated by mice that were deficient in cathepsin K [100] or *β*3 integrin [101]. Similarly, although c-Src-deficient mice had normal numbers of OCs, they developed osteopetrosis because of the inability to constitute ruffled membranes [102]. C-Src is therefore essential for the formation of ruffled membranes and for bone resorption [103]. Hence, the tyrosine kinase p60 c-Src may play roles in the translocation and/or fusion of intracellular vesicles by phosphorylating proteins that govern vesicular movements [103, 104]. Accordingly, endocytic proteins such as Rab11 may control c-Src subcellular localization [105].

In humans, three mutations have been shown to interfere with osteoclastic bone resorption. Specifically, these mutations were identified in* CAII*, which encodes a carbonic anhydrase that catalyzes hydration of carbon dioxide to produce carbonic acid and protons [106],* TCIRG1*, which encodes the alpha subunit of a proton pump V-ATPase, and* ClC-7*, which encodes a CCL-7 chloride channel that works with V-ATPase to acidify bone while maintaining electroneutrality [107]. In addition, loss-of-function mutations in the* PLEKHM1* gene caused an intermediate form of osteopetrosis in humans, with no or underdeveloped ruffled membranes in patient-derived OCs [90], potentially reflecting cessation of interactions with Rab7 in late endosomal/lysosomal vesicles.

### 7.2. Paget's Disease of Bone

Paget's disease of bone (PDB) is characterized by focal and disorganized increases in bone turnover. Because the initial phase of PDB involves excessive bone resorption, impaired OCs have been considered the primary cellular consequence of PDB [108]. Pagetic OCs are larger and more numerous than normal OCs, are overactive and hypersensitive to osteoclastogenic factors, and are resistant to apoptosis [109]. Inclusion bodies in OCs of affected bone are a well-described pathognomonic feature of PDB, and these aggregates of misfolded or ubiquitinated proteins contained p62 and ubiquitin [110]. Hence, because inclusion bodies in pagetic OCs resemble the p62 aggregates observed in diseases of defective autophagy, the pathogenesis of PDB likely reflects impaired autophagy [110]. In our previous study, defects in autophagy flux were observed in PBD OCs, suggesting an accumulation of nondegradative autophagosomes [53]. Activation of TBK1 (TANK binding kinase1) and TBK1-induced IL-6 production may also contribute to the generation of PDB OCs [111]. Interestingly, Rab8B has been shown to recruit TBK1 to autophagic organelles and contributed to autophagy-mediated antimicrobial defenses, such as autophagic elimination of mycobacterium tuberculosis, by phosphorylating and activating p62 [55, 112].

Although a viral etiology has been suggested for PDB, several studies reveal genetic components with marked effects [113–115]. Accordingly, mutations of the p62 gene* SQSTM1* are an important cause of PDB and the p62^P392L^ mutation contributed to overactivity, resistance to apoptosis, and basal NF-*κ*B activation in pagetic OCs [109, 116]. Hence, other genetic factors may remain unknown and will likely account for the vast majority of cases that are not caused by* SQSTM1* mutations. In addition to mutations and changes in gene expression, specific RNA isoforms of OC-related genes may contribute to overactivity of pagetic OCs. In particular, alternative splicing (AS) plays significant roles in protein diversity and in post-transcriptional gene regulation. However, despite the promise of biological relevance [117], the effects of AS isoforms in bone cells remain elusive. In a previous study, we identified AS events in the* TBC1D25* gene, which encodes TBC1 domain family member 25 (TBC1D25), and demonstrated that it is expressed in human OCs. Specifically, in the analyses of the two spliced isoforms of* TBC1D25,* we found a slight but significant decrease in mRNA and protein expression of the long isoform in pagetic OCs compared with controls, and these observations were independent of* SQSTM1 *mutations [40]. These data suggest that AS regulates the proportion of active TBC1D25 and, among known OC-expressed Rab proteins, Rab13, Rab33B, and Rab34 may interact with TBC1D25 [69, 118]. Rab33B is a substrate of TBC1D25 that plays roles in the maturation of autophagosomes and interacts with Atg16L1 [69] and is involved in protein transport from Golgi complexes to the ER [119]. Because Rab34^+^ vesicles fuse with autophagosomes [120], Rab34 may also be involved. Moreover, in OC precursors (RAW264.7 macrophages), Rab34 regulates lysosome localization to peripheral or central cellular compartments [121], and the Rab7 effector protein RILP, which has been associated with lysosome positioning, was recently identified as a Rab34 effector protein [121]. In polarized cells such as OCs, TBC1D25 may affect the formation of ruffled borders and consequently bone resorption, in addition to having roles in autophagy.

Finally, RIN3 is a GEF for the small GTPases Rab5 and Rab31 and has been associated with endocytosis, vesicular trafficking, and signal transduction. Although the role of RIN3 in bone metabolism has not been studied specifically, the* RIN3* gene is a reported predisposing factor for PDB [39].

### 7.3. Rab Small GTPases as Therapeutic Targets

Bisphosphonates are anticatabolic drugs that are widely used to treat diseases of increased bone resorption, such as PDB, osteoporosis, and malignant osteolysis. These agents inhibit bone resorption, reduce fracture risk in osteoporosis, and prevent skeletal events such as osteolysis and hypercalcemia in malignant bone diseases. Bisphosphonates directly suppress OC activity and predominantly induce OC apoptosis. Small GTPases, such as Ras, Rho, and Rab, are targets for nitrogen-containing bisphosphonates (N-BPs) that inhibit posttranslational prenylation [122]. N-BPs inhibit FPP synthase, a key enzyme in the mevalonate pathway, thus depleting cells of FPP and GGPP, which are required for the prenylation of small GTPases and are essential for their localization and function. Small GTPases are crucial signaling proteins that regulate various processes that are necessary for OC function, such as cytoskeletal organization, vesicular trafficking, and cell survival, and disrupted prenylation may result in OC apoptosis [123]. In addition, the antiresorptive effects of N-BPs may follow mislocated small GTPases, leading to dysregulation of cytoskeletal rearrangements and inhibited formation of ruffled borders during polarization of OCs. These morphological changes have been observed after treatments with N-BPs, and disrupted F-actin rings and the absence of ruffled borders were observed [14, 124].

Although N-BPs are more likely to affect geranylgeranyled small GTPases [14], they do not inhibit prenylation of Rab proteins specifically. Conversely, the N-BP analog phosphocarboxylate inhibits the mevalonate pathway enzyme RGGT, resulting in specific inhibition of Rab protein prenylation. Similarly, OCs from* gunmetal *mice have reduced RGGT activity but can form normally and polarize into sealing zones with no disruption of F-actin rings. These OCs exhibit reduced bone resorption activity because of impaired ruffled border formation* in vitro*. However, the remaining prenylated Rab proteins were sufficient to maintain normal bone resorption* in vivo*. Moreover, Rab7 protein remained 86% prenylated in these mice, and not all Rab proteins were affected by the reduction in RGGT activity [75]. Although prenylation is essential for the localization of Rab GTPases to specific intracellular compartments, targeting prenylation may not be sufficient to alter functions in OCs. In addition, OCs expressing the prenylation-deficient Rab3D had similar resorptive activity as those with constitutively active GTP-bound Rab3D and wild-type cells, and only OCs expressing constitutively inactive Rab3D (GTP-binding deficient) had impaired bone resorption capacity [74]. These data warrant further investigations of the roles of Rab proteins in crucial pathways of resorptive OC and on the rate-limiting steps (GTP/GDP bound or prenylation) that regulate their functions. Potentially, targeting specific Rab proteins in certain states may be a more efficient therapeutic option for some bone diseases.

## 8. Conclusion

Small GTPases of the Rab family play major roles in OC functions, particularly in autophagy and bone resorption through ruffled membranes. These proteins are involved in various vesicular trafficking pathways in OCs and may become therapeutic targets for bone diseases. Mechanisms of lysosome trafficking and autophagy that involve Rab proteins in OCs may be unique to this secreting cell type, and further studies of Rab proteins in OCs will be compelling. However, the determinants of tissue and cell type specificity of these small GTPases need to be identified [125].

Even if our knowledge increases in the understanding of the bone resorption process, important pieces of the puzzle are missing to understand the related trafficking phenomena, from the formation of secretory vesicles, to their transport and fusion to the ruffled membrane. It is obvious that other Rabs in addition to those already identified to date (Rab3D, Rab27A, and Rab7) are directly involved in the process. Determining the specific Rabs involved in each step of the secretory vesicle trafficking would be useful for improving our knowledge of osteoclast biology and bone diseases, to identify specific targets of the bone resorption process or to facilitate the study of bone resorption by identifying markers of secretory vesicles for bone resorption. In addition, the study of regulators and signals coordinating the involvement of Rab7 in either autophagy or bone resorption might help to clarify conflicting data on the impact of autophagy on bone resorption in OCs [54, 79].

## Abbreviations

Beclin 1: BCL-2 interacting myosin/moesin-like coiled-coil protein 1
DENN: Differentially expressed in normal and neoplastic cells
ER: Endoplasmic reticulum
FPP: Farnesyl pyrophosphate
FYCO1: FYVE-coiled coil containing 1 (protein)
GAP: GTPase-activating protein
GDI: GDP dissociation inhibitor
GDF: GDI displacement factor
GEF: Guanine–nucleotide exchange factor
GGPP: Geranylgeranyl pyrophosphate
HOPS: Homotypic fusion and protein sorting complex
LAMP2: Lysosome-associated membrane protein2
LC3: Light chain 3 [Atg8 (yeast) is called LC3 in mammals]
MCSF: Macrophage colony-stimulating factor
mTORC1: Mammalian target of rapamycin complex 1
OC: Osteoclast
ORP1L: Oxysterol-binding protein-related protein 1L
PDB: Paget's disease of bone
Phafin1: A FYVE and PH (Pleckstrin homology) domain containing protein
PLEKHF1: Pleckstrin homology domain-containing family F member 1 (aka Phafin1)
PLEKHM1: Pleckstrin homology domain containing, family M (with RUN domain) member 1
PTB and SH2 domains: pTyr-binding (PTB) and Src homology 2 (SH2) domains
PtdIns3P: Phosphatidylinositol 3-phosphate
RANKL: Receptor activator of NF-*κ*B ligand
REP: Rab escort protein
RGGT: Rab Geranyl-Geranyl transferase
RILP: Rab7-interacting lysosomal protein
RIN3: Ras and Rab Interactor 3
SNAREs: N-ethylmaleimide-sensitive factor attachment protein receptors
TAB1 and TAB2: TAK1 binding protein 1 and 2
TAK1: TGF*β*-activated kinase 1
TBC: Tre-2/Bub2/Cdc16
TBK1: TANK binding kinase1
TRAF6: TNF receptor-associated factor 6
TRAPP: Transport protein particle
UBA: Ubiquitin-associated domain
ULK1: UNC51-like kinase 1
V-ATPase: Vacuolar H^+^-ATPase
VPS: Vacuolar protein sorting
WIPI: WD repeat domain phosphoinositide-interacting protein.

## Supplementary Materials

Supplementary Materials Supplemental Table: GTPases expressed in osteoclasts: summary of their regulators, effectors, and functions as identified in other cell types.
