# Supplementary material for: Rab GTPases in Osteoclastic Endomembrane Systems
Source: Biomed Res Int. 2018 Aug 15;2018:4541538. doi: 10.1155/2018/4541538 (PMC6114073; doi:10.1155/2018/4541538)
Supplement: Supplementary Materials — Supplemental Table: GTPases expressed in osteoclasts: summary of their regulators, effectors, and functions as identified in other cell types. [file 4541538.f1.pdf]

Supplemental Table: ***GTPases expressed in osteoclasts: Summary of their regulators, effectors and functions as identified in other cell types***

GTPase in OC	TBC/RabGAP	RabGEF	Effector protein	Primary trafficking function [26, 29, 33]	Other trafficking function
**Rab1B[14, 26, 72]**	TBC1D14[63], TBC1D20[37, 38, 55] ; USP6NL[38]	TRAPPI subunits (TRAPPC1, TRAPPC2, TRAPP2L, TRAPPC3, TRAPPC4, TRAPPC5, TRAPPC5A/B)[35]; TRAPPIII subunit TRAPPC8[63]; DrrA[24]	Golgin84[63]; p115[22, 29, 46]; GM130[29]	ER to Golgi trafficking	Autophagy[46, 57]
**Rab2B[75]**	TBC1D11[37, 38]; TBC1D20[37, 38]; USP6NL[38]	NI	Bicaudal-D[34,43] ; GM130/p115[43]; HOPS complex (subunit Vps39)[43]; golgin-45[43]	ER to Golgi trafficking	NI
**Rab3[26]A[72, 67]/B[72, 73]/C[31, 73]/D[14, 32, 74, 75]**	TBC1D10B (A)[37, 38]; USP6NL (A)[38]; RAB3GAP*[34] (A)[118]	MADD[35]; Rab3GEP[118]; GRAB[35, 118]	Rabphilin3[22, 34]; Noc2[22]; RIM1[22]; RIM2[22]; SNAP29 (A)[56]	Exocytosis; Golgi to apical membrane trafficking	Secretory granules[74]; Bone resorption (D)[31, 32, 74, 75]
**Rab4A[31]/B[14, 25, 72]**	TBC1D11[37, 38]; RUTBC3[118]; EVI5L (A)[38]	NI	Rabaptin4[22]; Rabaptin5[22, 33]; Rabip4[43]; Rabaptin5β[22]; GARP complex (Vps51, Vps52, Vps53)[43]; Rabenosyn5[34]	Early/recycling endocytosis	Autophagy[55, 57]
**Rab5A[31]/B[31]/C[14, 26, 72]**	TBC1D3[37, 38, 118]; RUTBC3[118] (A-C)[37, 38]; USP6NL[37, 118] (A)[38]	RIN1[35, 55, 118]; RIN2[35, 55, 118]; RIN3[35, 39, 55, 118]; RINL[35]; ALS2[35, 55, 118]; ALS2CL[35, 55]; Rabex5[29, 34, 55, 118]; Vsp9[55]; GAPVD1[35, 55]	HOPS complex[34]; Rabenosyn5[22, 43]; EAA1[29, 72]; p150[22]; Vac1[22, 55]; PI3K[22]; Vps34 [43, 55]; Vps45[22]; CORVET[22, 55]; Rabaptin5 [29, 34, 43]; huntingtin[43]; Kif13A[43]; Ccz1[43]; TBC1D15[43] ; APPL[29]	Early endocytosis	Autophagy[46, 55, 57, 64]; Macropinocytosis[38]
**Rab6[26, 75]**	TBC1D11[37, 38, 118]	RIC1-RGP1[29, 35]	Rabkinesin6[22]; GAPCenA[22]; OCRL1[43]; TMF1[43]; EVI5[43]; TRAPPII[43]; DENND5[43](A)[34]; TBC1D5[43]; Bicaudal-D[34, 43, 56]	Intra-Golgi trafficking	NI
**Rab7[15, 31, 72, 75, 84, 89, 90, 91]**	TBC1D2[37, 38, 85] ; TBC1D5[37]; TBC1D15[37, 38, 55,118] ; RUTBC3 [118] ; EVI5L[38]	Mon1-Ccz1[23, 24, 29, 35, 55] ; Vsp39[23] ; HOPS complex[34]	RILP[55, 23, 84, 90];ORP1L[23, 55;] Rabring7[22, 90]; Rac1[90, 92]; PLEKHM1[84, 91]; HOPS complex[22, 23] (subunit Vps39, Vps41)[29]; FYCO1[23,46, 55] PLEKHF1 (Phafin1)[55]; VAMP8[55]; Rubicon[43]	Late endocytosis	Autophagy[46, 55, 56]; Bone resorption[23, 31, 32, 90, 91, 92]
**Rab8B[31]**	TBC1D1[38]; TBC1D17[55]	RABIN8[24, 35, 55, 118]; RPGR[35]; GRAB[35]	Rab8IP[22]; TBK1[46, 55]; Sec15[35]	Golgi to basolateral membrane trafficking	Xenophagy[46, 55, 57]; Wnt/β-catenin signaling[46]
**Rab9[14, 26, 72]A[31]**	NI	DENND2A[31, 55]; DENND2B[35]; DENND2C[35]; DENND2D[35]	HSP4[35]; p40[22] (A)[29]; TIP47[22] (A)[29]; RUTBC1[29, 34]; GM130[43]; RUBTBC2[29, 34]	Late endocytosis to *trans* Golgi	Xenophagy[46, 55]; Cholesterol trafficking[30]
**Rab10[26, 31. 75]**	TBC1D1[38]; TBC1D4[37, 38]; RUTBC3[118]; EVI5L[38, 118]; RAB3GAP*[118]	DENND4A[35]; DENND4B/C[35]	EVI5[37]; EVI5L[37]	Golgi to basolateral membrane trafficking	NI
**Rab11[15, 75]A[31]/B[14, 26, 72]**	TBC1D11[37, 38]; TBC1D15[38] (A)[37]; EVI5[37, 38, 55]	TRAPPC2[55]; HTT complex[55]; REI1[35]	TBC1D14[46, 55, 63]; Myosin Vb (A)[34]; Rabin8[35]; Rabiphilin11[22]; Rab11BP[22]; FIP2[22]; FIP3[22]; FIP4[22]; Sec15[22, 35]; RIP11[22]; PI4KIIIβ (A)[34]	Recycling endocytosis	Autophagy[46, 55, 57, 64]
**Rab12[31]**	NI	DENND3[35, 42]	RILPL1[35, 42]	Vesicles transport from peripheral to perinuclear region	Autophagy[64]
**Rab13[31, 32]**	TBC1D25[38, 40]	DENND1C[35]; DENND2B[35]	MICAL-L2[35]; Delta-PDE[22]	NI	Tight junctions assembly[31]; Traffic of glucose transporters[31]; Neuron regeneration[31]
**Rab14[31, 75]**	TBC1D1[38]; TBC1D4[37, 38]	DENND6A/B[35], FAM116A[29]	NI	Transport between endocytosis and Golgi	NI
**Rab18[31, 75]**	NI	RAB3GAP1-RAB3GAP2[35, 43, 65]	Syntaxin-18[43]; TBC1D5[43]; LRRK2[43]; Spartin[43]; NRZ complex[43]	ER to Golgi trafficking	Autophagy[64]; Virus infection [65]; Lipid droplets metabolism[43, 65]
**Rab20[31]**	NI	NI	NI	Recycling/apical endocytosis	Vacuolar H^+ ATPase trafficking[30]
**Rab21[31]**	TBC1D17[37, 38]	MTMR13[70]; Rabex5 [35]; RINL[35]; Varp[29, 70]	VAMP8[70]	Early endocytosis	Autophagy[70]
**Rab22A[31]**	TBC1D10B[37, 38]; TBC1D18[37, 38, 118]; RAB3GAP*[118]	Rabex5[35]; RINL[35]	TBC1D2B[37]; Rabenosyn5[34]; EEA1[34]	*Trans* Golgi network to endocytosis	Phagosome maturation (A)[30]
**Rab23[31]**	EVI5L[37, 38]	NI	NI	Transport between plasma membrane and endocytosis	Xenophagy[46]; Hedgehog signaling[30, 46]
**Rab24[31]**	NI	NI	NI	NI	Autophagy[46, 56, 57]; Cell division[46]
**Rab27A[31, 32, 46]/B[32]**	TBC1D10A (A)[37, 38]; TBC1D10B (A)[37, 38]	MADD[35, 46]	Melanophilin[101] (A)[34]; Mun13-4 (A)[34]; Rabphilin3[22]; Noc2[22]; Granuphilin[22]; SIp2a[34]	NI	Secretory granules[32]; Melanosome transport[32]; Bone resorption (A)[32]
**Rab30[31]**	NI	NI	p115[43, 57]; Bicaudal-D[43,57]; GM130[43]; Iκ2[43]; USP6NL[37]	NI	Xenophagy[57]
**Rab31[31]**	TBC1D10B[38]	GAPVD1[35]; RIN1[35]; RIN2[35]; RIN3[35, 39]; RINL[35]; ASL2[35]; ALS2CL[35]	NI	*Trans* Golgi network to endocytosis	NI
**Rab32[31]**	RUTBC1[29, 34, 55]	HPS1-HPS4[29, 34, 35]	Varp[35]	NI	Autophagy[46, 55, 64]; Mitochondrial dynamics[31, 46]; Melanosome transport[31, 46]
**Rab33A[31]/B[31]**	TBC1D25[38, 40, 69]; RUTBC1 [34]	NI	Rab33B-BP[22]; Rabaptin5[34]; Atg16L[68]	Intra-Golgi trafficking	Autophagy (B)[46, 55, 57, 68, 69]
**Rab34[31]**	TBC1D18[38, 118]; TBC1D25[38, 40, 118]	NI	RILP[22, 90]	NI	Macropinocytosis[30], Lysosomes distribution[30]
**Rab35[14, 26, 72]**	TBC1D10A[37, 38, 58]; TBC1D10B[37, 38]; TBC1D20C[37, 38]; TBC1D17[37]; EVI5[37, 38]	DENND1A[35]; DENND1B[24, 35]; DENND1C[35]; FLNC[35]	NI	Recycling endocytosis	Sorting melanosomes[32]; Phagosome maturation[32]; T cell recycling[38]; Mitophagy[58]
**Rab38[31, 77]**	NI	HPS1-HPS4[29, 34, 35]	Varp[35]	NI	Melanosome transport[22, 32, 35]; Secretory granules[22]
**Rab44[78]**	NI	NI	NI	NI	NI

*Underlined: Expressed or function in OCs; Superscript: Reference; (X): Specific to the isoform; *Atypical RabGAP without TBC domain; NI: Not identified*
